# Exploring relationships between dating app use and sexual activity among young adult college students

**DOI:** 10.3389/frph.2024.1453423

**Published:** 2024-11-15

**Authors:** Jaquetta M. Reeves, Stacey B. Griner, Kaeli C. Johnson, Erick C. Jones, Sylvia Shangani

**Affiliations:** ^1^ College of Nursing and Health Innovation, University of Texas at Arlington, Arlington, TX, United States; ^2^ University of North Texas Health Science Center, School of Public Health, Fort Worth, TX, United States; ^3^ Industrial, Manufacturing, and Systems Engineering (IMSE) Department, University of Texas at Arlington, College of Engineering, Arlington, TX, United States; ^4^ Department of Community Health Sciences, Boston University, School of Public Health, Boston, MA, United States

**Keywords:** youth, dating apps, sexual behaviors, college & university students, STI/HIV testing

## Abstract

**Background:**

University campus clinics provide crucial sexual health services to students, including STI/HIV screening, testing, contraception, and counseling. These clinics are essential for engaging young adults who may lack access to primary care or have difficulty reaching off-campus services. Dating apps are widely used by young adults, yet there is a lack of studies on how they affect sexual practices. This study aimed to evaluate the use of dating apps, engagement in condomless sexual activity, and the prevalence of STIs among young adult college students in Northern Texas.

**Methods:**

A cross-sectional survey was conducted from August to December 2022 among undergraduate and graduate students aged 18–35 at a large university in Northern Texas. A total of 122 eligible participants completed the survey, which assessed demographics, sexual behaviors, dating app use, and STI/HIV testing practices. Descriptive statistics, bivariate analyses, and multivariate Poisson regression analyses with robust variance were performed to identify factors associated with dating app use and condomless sexual activity.

**Results:**

Two-thirds of participants reported using dating apps. Significant differences were found between app users and non-users regarding demographic factors and unprotected sexual behaviors. Dating app users were more likely to report multiple sexual partners, inconsistent condom use, and a higher likelihood of engaging in unprotected sex. Poisson regression analysis indicated that app use was associated with residing in large urban areas, frequent use of campus STI/HIV screening services, and having multiple sexual partners (*p* < 0.05).

**Conclusion:**

The study highlights a significant association between dating app use and engagement in condomless sexual activity among college students. University health clinics play a critical role in providing sexual health services and can enhance their impact by expanding access to testing, contraception, and inclusive, sex-positive education. Future research should explore the long-term effects of dating app use on sexual health and evaluate the effectiveness of app-based interventions in promoting safer sexual practices.

## Introduction

Geosocial networking applications, commonly known as “dating apps,” have existed for over 20 years but began to gain significant traction around 2010 (1). Since then, the prevalence of these apps has surged among young adults (2–4), primarily as platforms facilitating immediate sexual encounters where users can select partners based on specific characteristics (1, 4, 5). Research has shown that dating apps have made it easier for users to find partners for casual sex or “hooking up” (2, 6, 7). For example, one study found that app users were twice as likely to have engaged in unprotected sexual activity within the previous three months compared to non-users (8). Another study reported that the use of dating apps was associated with having three or more sexual partners, hookups, and condomless sex (9). Furthermore, research has found a link between the use of online dating apps and adverse psychological effects, including depression and anxiety, having a negative impact on a person's emotional health (10–12). Some studies suggest that motivations for using dating apps, such as seeking validation or ease of communication, are positively associated with eating disorders. In contrast, motivations like seeking excitement or love are negatively associated (13, 14). Other studies identified several primary motives for using these dating app platforms, such as entertainment and adventure by browsing individuals’ profiles, interacting and forming relationships with others, comfort in reduced pressure of digital communication vs. face-to-face interactions, and anonymity makes it easier for users to express a desire for hookups (e.g., casual sexual encounters). These studies also highlight how attitudes toward cyber dating, self-esteem, age, and gender influence these motives (15–17). Given these findings, it is unsurprising that there has been an observed increase in sexually transmitted infections (STIs) and HIV infections among individuals aged 15–24 years of both sexes (18). Despite these associations, there is still limited empirical evidence to directly link dating app use with an increased risk of acquiring STIs and HIV among young adults.

Transmission of STIs and HIV continues to be a major public health issue in the United States (7). Between 2015 and 2019, the Centers for Disease Control and Prevention (CDC) reported a rise in HIV diagnoses among individuals aged 13–24 years. Approximately 51% of young people living with HIV were unaware of their infection (7, 18). Furthermore, current national surveillance statistics reveal that within this age group, over 20% of new HIV diagnoses are attributed to hazardous sexual activities (19). Engagement in condomless sexual behavior can result in negative consequences such as the transmission of STIs, HIV, and unintended pregnancies (19). RSB are defined as (1) engaging in oral, vaginal, or anal intercourse without using a condom (whether male, female, or dental dam) or having irregular condom usage, (2) engaging in sexual relationships with multiple partners or a high-risk partner (one who has multiple sex partners or other risk factors, such as intravenous drug use), (3) initiating sexual activity at an early age, especially before the age of 18, (4) frequently changing sexual partners, (5) having the status of “friends with benefits” without any commitment, and (6) engaging in casual sexual encounters (7, 19–22).

In this study, a “sex partner” is defined as any person with whom an individual has participated in sexual activity, encompassing vaginal, oral, or anal intercourse, irrespective of the length, frequency, or emotional bond established in the relationship (20). “Friends with benefits” (FWB) refers to a connection between two individuals who sustain a friendship while participating in sexual activities without the expectations or commitments characteristic of a romantic union (21). Hook-up sex refers to sexual activity that takes place outside of a committed, love relationship, typically characterized by spontaneity and a casual atmosphere (21, 22). Typically, this form of sexual contact has minimal emotional bonding or anticipation of future commitment. Hook-ups can occur among strangers, acquaintances, friends, or “friends with benefits,” and encompass a variety of sexual engagements, ranging from kissing and physical contact to different types of intercourse (21, 22). The incentives for participating in hook-up sexual activity can be extensively diverse, including physical gratification, exploration, social cohesion, or reaction to peer pressure (22).

Young sexually active individuals should be tested for HIV at least annually, and individuals who are at a higher risk of contracting HIV should be tested annually, depending on their sexual habits (18). However, a significant proportion of young adults do not receive the recommended sexual health services, including screening and counseling for STIs and HIV. This can be attributed to various barriers such as restricted availability of STI/HIV preventive services, inadequate insurance coverage or lack thereof, concerns about disclosing their utilization of services to their parent's insurance, and limited transportation options (23–25). Due to confidentiality concerns, costs, embarrassment, shame, and other social factors, many adolescents and young adults do not proactively seek STI and HIV testing with their primary care providers (26). The CDC national survey showed that STI and HIV testing guidelines among youth aged 15–25 are not regularly followed, and STI testing is suboptimal (26, 27). In 2019, new HIV diagnoses were highest among people aged 25 to 29, with the Southern regional areas continuing to see the highest rates of new cases (18, 19). Consequently, those who are infected with an STI, or HIV may transmit infections to their partners without awareness of their status (18, 19). Several studies focus on dating app usage, but findings may not apply to young college students. These studies often cover a wider age range, including individuals in various life stages (3, 6, 28–30). In contrast, college students who are undergraduates between the ages of 18−24 are going through a unique growth stage marked by self-discovery, autonomy, and trying new social experiences (31). This unique stage may impact their motives and actions differently compared to older young adults, who may be working, married, or parenting (32). Research studies conducted in other countries have shown a correlation between the use of mobile dating apps by university undergraduate students and engaging in condomless sexual behaviors. Lindley et al. (2017) found that dating apps like Tinder provide opportunities for social interaction and sexual identity exploration but can lead to risks of having unprotected sex and more than one sexual partner, particularly among young adult college students (33). Choi et al. (2016) found that dating apps are popular for social connections but also linked to increased unprotected sexual behaviors, such as condomless sex with a casual partner (5). These findings highlight the need for increased awareness and prevention of condomless sexual behaviors among college students.

Moreover, some studies, particularly those focused on adult men who have sex with men (MSM), may not capture the experiences or behaviors of heterosexual college students (2, 5, 6, 9, 14–17, 28, 30, 34). Because of these differences in demographics, motivations, social contexts, app usage patterns, cultural influences, psychological factors, and technology use, previous studies focusing on broader young adult populations, particularly those involving specific subgroups like MSM, may not be directly applicable to heterosexual college students (32). Understanding these distinct characteristics is crucial for developing interventions, educational programs, and research that accurately address the needs and behaviors of college student populations.

University campus clinics are crucial in addressing these issues as they offer students accessible, private, and affordable sexual health services (35–37). University health clinics are in a special position to engage young adults in sexual health services, especially because numerous college students may not have existing connections with primary care providers or may struggle to reach health services off campus (35). These establishments typically provide a variety of services such as STI/HIV screening and testing, contraception, and sexual health counseling, crucial for detecting, preventing, and early treatment (35, 37). Campus clinics can also utilize focused interventions like sexual health education campaigns, peer-led workshops, and collaborations with student groups to increase awareness and access to sexual health services (35). These initiatives can be highly successful in reaching students who are not actively seeking treatment because of false information, limited knowledge, or cultural factors (35). University campus clinics are essential for improving sexual health outcomes and decreasing transmission rates among young adults, particularly in areas with high rates of HIV diagnoses in this age group (35–37). The objective of this study was to evaluate the usage of dating apps, engagement in condomless sexual activity, and the prevalence of STIs among young adult college students in Northern Texas.

## Methods

### Study design, population and data collection

A cross-sectional survey was conducted using QuestionPro to administer an anonymous online questionnaire from August 28, 2022, to December 20, 2022. The study targeted undergraduate and graduate students aged 18 to 35 enrolled at a large university in Northern Texas. A total of 434 students were invited to participate, with 152 responding to the survey, resulting in an 82% completion rate. Of these, 28 students withdrew from the survey, and 2 were excluded for not meeting the age criteria, leaving 122 eligible participants.

Participants were recruited via an email distribution sent by the Department of Communications and flyers posted across the campus containing a QR code for survey access. To be eligible for the study, participants needed to be 18 years or older, sexually active, able to read English, and currently enrolled as students. The survey automatically terminated if respondents did not meet these eligibility criteria.

Following informed consent, eligible participants completed a 10–12-minute online survey. To ensure data integrity and prevent multiple submissions, we used QuestionPro, which employs several safeguards, including tracking IP addresses, setting browser cookies, and utilizing digital fingerprinting technology to identify unique devices. These measures restrict participants to a single survey submission and flag any attempts at duplicate entries. At the end of the survey, participants had the option of going to a separate webpage and entering their email address to be included in a raffle drawing for one of the six $50 Amazon electronic gift cards. For anonymity, names and email addresses were not connected to participants’ survey responses. The university's Institutional Review Board granted a waiver of informed consent.

### Measures

Demographic variables collected included age, sex, race, marital status, academic status (undergraduate or graduate), and area of residence, defined by the participant's permanent home address (categorized as either large urban or small town). The dependent variable was a binary measure of dating application use, assessed by asking participants if they had ever used a dating app, with responses recorded as “yes” or “no.”

All participants were asked about their sexual activities, including the number of sexual partners, HIV-testing patterns, condom use, and age at first sexual encounter. The survey measured STI and HIV exposure, testing, and treatment using the CDC's 5 P's of sexual health history taking (partners, practices, protection from STIs, history of an STI, and pregnancy prevention) (38). The 5 P's are the latest clinical guideline using open-ended questions to assess risk-taking sexual behaviors (i.e., condomless sex, multiple sex partners, uncommitted sex partners), and information on sexual orientation and gender identity history components (38). We assessed the consistency of condom use with sexual partners. An example of this assessment includes “How often do you use condoms for vaginal, anal, or oral sex?” The response options ranged from 1 (always) to 5 (never). For the analysis, we trichotomized variables to (always/often = 1, sometimes = 2, and rarely/never = 0). Attitudes toward condom use were measured using the Attitudes Toward Condoms (ATC) Scale (39), a 5-point Likert scale comprising 41 items. The five options on the scale were as follows: 1 = always, 2 = often, 3 = sometimes, 4 = rarely, 5 = never, 1 = strongly agree, 2 = agree, 3 = disagree, and strongly disagree = 1. The ATC instrument was developed to measure the degree of favorableness or lack thereof toward the personal use of contraception (i.e., condom use) during sexual activity (40). The original ATC scale has a Cronbach alpha score of 0.93 (39). A Cronbach alpha score of 0.92 was obtained for this current pilot indicating that the instrument had a high internal consistency. Measures do not reference a specific recall period, with the exception of number of sexual partners, which is based on recall over the past year. The survey included three fill-in-the-blank questions that asked respondents about their sexual partners using dating apps. Dating app use referred to ever use of the app not current use. The participants were also asked about their use of university students’ health services. Two questions required binary responses (“Yes” or “No”) developed to assess knowledge and use of the university student health services.

### Data analysis

Descriptive statistics were used to characterize the participants. Frequencies and means were calculated for the sample demographics and sexual risk factors, followed by bivariate analysis. We categorized variables with skewed distribution of the data such as age and number of sexual partners. We conducted bivariate analyses using chi-square and *t*-tests to analyze the significant associations between dating apps and demographic and sexual risk factors. In addition, these analyses identified differences between participants who used dating apps and those who did not use dating apps across the various independent variables. Multivariate Poisson regression analyses with robust variance were conducted to identify unprotected sexual behaviors associated with the use of dating apps. Potential confounders–variables that have been previously found to be associated with dating app use and sexual behavior such as condom use, frequency of STI/HIV testing, and multiple sexual partners were used in the final logistic regression model. In addition, we used factors that were statistically significant at a *p*-value of 0.05 in the final regression model. Prevalence ratios (PRs) and the corresponding 95% confidence intervals (CI) are presented. IBM SPSS Statistics for Windows (2020), version 27.0, was used for all the analyses.

## Results

### Demographics

The sample comprised of 122 participants, as shown in Table 1A. Of 122 respondents, 45% were white. The mean age of the participants was 23 (SD = 3.5) years. More than half of the participants were male (54.6%), and the majority were heterosexual (79%). Most were single (77.5%), undergraduates (77%), were aware of campus-based student health clinic services (83%) and resided in small towns as their permanent home address (64%). Over half of the participants were 18−24 years old (63%).

**Table 1A:** Characteristics of study participants (*N* = 122) at a large university in Northern Texas, USA, 2022.

Characteristics	*N* (%) or Mean ± SD
Age M(±SD)	23.3 (3.5)
18–24	77 (63.1)
25–35	45 (36.9)
Race	
Black	12 (9.8)
Hispanic/latino	16 (13.1)
Other	39 (32.0)
White	55 (45.1)
Gender identity	
Male	66 (54.6)
Female	53 (43.44)
Other (transgender and transsexual)	3 (2.46)
Sexual orientation	
Heterosexual	96 (79.3)
Gay/lesbian	10 (8.26)
Other (bisexual and other)	15 (12.40)
Relationship status	
Married/with partner	27 (22.5)
Single	93 (77.5)
Academic program	
Undergraduate	77 (77.0)
Graduate	23 (23.0)
Area of residence	
Large urban	58 (47.5)
Small town	64 (52.5)

Race Other: American Indian/Alaskan Native, Middle Eastern, Asian, not identifying as Black, White, and Hispanic/Latino. Gender identity other—Transgender and transexual, sexual orientation other. Due to missing data, some totals do not add up to 100%.

### Unprotected sexual behaviors

Regarding condomless sexual behaviors (Table 1B), two-thirds of participants used dating apps (67%). More than half reported that they had received HIV and STI screening services at a student clinic (53%). More than one-third of the participants (34%) reported that they worried about pregnancy, one-third worried about STIs, and close to 30% worried about both STIs and pregnancy. Almost all participants reported ever having oral, anal, and vaginal sex (98%), with more than half reporting first sexual encounter between the ages of 16-19 years (55%). The majority of the participants reported ever testing for HIV and STI (69%), having more than one sexual partner (71%), and frequently testing for STI in less than six months (63%). More than one-third of participants reported sex while drinking alcohol (35%). On average, attitude toward condom use was 28.5 (SD = 5.2) on a 13-item scale that ranged from 1 to 52 (Table 1B).

**Table 1B:** Sexual behavior sample characteristics of participants (*N* = 122).

Characteristics	N (%) or Mean ± SD
^a^Dating app use	
Yes	66 (66.70)
No	33 (27.05)
Aware of student health clinic (yes)	100 (82.6)
Ever received HIV/STI screening at student clinic (yes)	65 (53.2)
Worried about Pregnancy or STI	
Pregnancy	41 (33.6)
STI	39 (32.0)
Both	33 (27.0
None	9 (7.4)
Ever had sex (oral, anal, or vaginal) Yes	119 (97.5)
Age (years) at first sexual activity	
≤15	38 (31.4)
16–19	66 (54.6)
≥20	17 (14.1)
Sexual partners in the past year	
0–1	35 (28.9)
>1	86 (71.1)
Lifetime sexual partners	
1–2	44 (36.4)
3–4	44 (36.4)
≥5	33 (27.2)
Ever had STI and HIV testing (yes)	84 (68.8)
Last HIV test	
≤1 year	61 (50.4)
>1year	60 (49.6)
Ever tested positive for STI and HIV (Yes)	36 (29.5)
Frequency of STI testing	
Never	23 (18.8)
<6 months	77 (63.1)
>6 months	22 (18.0)
Sex while drunk (Yes)	43 (35.3)
Condom use frequency—vaginal	
Always/often	28 (22.9)
Sometimes	35 (28.7)
Rarely/never	59 (48.4)
Condom use frequency—oral	
Always/often	58 (47.5)
Sometimes	33 (27.1)
Rarely/never	31 (25.4)
Condom use frequency—anal	
Always/often	28 (23.1)
Sometimes	58 (47.9)
Rarely/never	35 (28.9)
Condom use consistency with sex partner M ± SD	9.0 (3.9)
Condom use consistency with friends M ± SD	9.4 (3.9)
Condom use consistency with hook-up sex M ± SD	10 (3.9)
Power over condom use	
Self	15 (12.4)
Partner	34 (28.1)
Both	47 (38.8)
Neither	25 (20.7)
Difficulty discussing condom use -Yes	20 (16.7)
Attitudes toward condom use^b^ M ± SD	28.5 (5.2)

Condom use consistency—sum, higher values mean higher self-reported frequency of condom use.

^a^ Scale variable, 13 variables, range 1–52.

^b^ Totals do not add up to 100% due to missing data.

### Differences between dating app users and non-app users

Bivariate analyses (Table 2) indicated significant differences between those who ever used dating apps and those who did not use dating apps. White students used dating apps more than any other group (47%). Only 12% of Black students in the sample used dating apps. Men were more likely to use dating apps than women (64% vs. 33%, *p* < 0.001). Participants who were identified as heterosexual were more likely to use dating apps than those who were identified as gay/lesbian (86% vs. 14%, *p* < 0.001). Participants who resided in large urban settings used dating apps significantly more frequently than those who resided in small-town settings (62% vs. 38%, *p* < 0.001). College students who reported ever receiving HIV or STI screening at the student clinic reported using dating apps more frequently than students who had never received HIV or STI screening at the student clinic (67% vs. 33%, *p* < 0.001). In addition, participants who reported their first sexual encounter at ages 16-19 were more likely to use dating apps than those who reported their first sexual encounter at ages 15 and below and 20 or older (51% vs. 28% vs. 11%, *p* < 0.01).

**Table 2:** Bivariable analysis of demographic and sexual behavior characteristics by dating app use among participants (*N* = 122).

Variables	Dating App Users *n* (%)	Non-dating app users *n* (%)	*P*-value
Age			0.87
18–24	45 (68.2)	22 (66.7)	
25–35	21 (31.8)	11 (33.3)	
Race			**0** **.** **001**
Black	8 (12.1)	1 (3.0)	
Hispanic/latino	1 (1.5)	13 (39.4)	
Other	26 (39.4)	9 (27.3)	
White	31 (47.0)	10 (30.0)	
Gender identity			**0**.**002**
Female	22 (33.4)	23 (69.7)	
Male	42 (63.6)	10 (30.3)	
Other	2 (3.03)	0 (0.00)	
Other (transgender and transexual)			
Sexual orientation			**0**.**07**
Heterosexual	56 (86.2)	23 (69.7)	
Gay/lesbian	5 (7.7).8)	3 (9.1)	
Other (bisexual and other)	4 (6.1)	7 (21.2)	
Relationship status			0.88
Married/with partner	13 (20.0)	7 (21.2)	
Single	52 (80.0)	26 (78.8)	
Academic program			0.88
Undergraduate	46 (82.1)	21 (80.8)	
Graduate	10 (17.9)	5 (19.2)	
Area of residence			
Large urban	41 (62.1)	8 (24.2)	**<0**.**001**
Small town	25 (37.9)	25 (75.8)	
Aware of services at student health clinic			0.55
Yes	52 (80.0)	28 (84.8)	
No	13 (20.0)	5 (15.2)	
Ever received HIV/STI screening at student clinic			**<0**.**001**
Yes	44 (66.7)	8 (24.2)	
No	22 (33.3)	25 (75.8)	
Worried about pregnancy or STI			**0**.**05**
Pregnancy	23 (34.8)	5 (15.2)	
STI	25 (37.9)	11 (33.3)	
Both	16 (24.2)	14 (42.4)	
None	2 (3.1)	3 (9.1)	
Sexual behaviors			
Age (years) at first sexual activity			**0**.**01**
≤15	25 (37.9)	4 (12.5)	
16–19	34 (51.5)	19 (59.4)	
≥20	7 (10.6)	9 (28.1)	
Sexual partners in the past year			**<0**.**001**
0–1	13 (19.7)	21 (65.6)	
>1	53 (80.3)	11 (34.4)	
Lifetime sexual partners			**<0**.**001**
1–2	18 (27.3)	23 (71.9)	
3–4	26 (39.4)	4 (12.5)	
≥5	22 (33.3)	5 (15.6)	
Ever had STI and HIV testing			**0**.**004**
Yes	51 (77.3)	16 (48.5)	
No	15 (22.7)	17 (51.2)	
Last HIV test			**<0**.**001**
≤1year	41 (63.1)	8 (24.2)	
>1 year	24 (36.9)	25 (75.8)	
Ever tested positive for STI and HIV			**0**.**06**
Yes	24 (36.4)	6 (18.2)	
No	42 (63.6)	27 (81.8)	
Frequency of STI testing			**<0**.**001**
Never	5 (7.6)	14 (42.4)	
<6 months	47 (71.2)	12 (36.4)	
>6 months	14 (21.2)	7 (21.2)	
Sex while using alcohol			
Yes	26 (39.4)	6 (18.2)	**0**.**03**
No	40 (60.6)	27 (81.8)	
Condom use			
Condom use frequency—vaginal			0.13
Always/often	20 (30.3)	4 (12.1)	
Sometimes	14 (21.2)	9 (27.3)	
Rarely/never	32 (48.5)	20 (60.6)	
Condom use frequency—oral			0.12
Always/often	30 (45.5)	22 (66.7)	
Sometimes	16 (24.2)	6 (18.2)	
Rarely/never	20 (30.3)	5 (15.2)	
Condom use frequency—anal			**<0**.**001**
Always/often	23 (34.8)	3 (9.1)	
Sometimes	19 (28.8)	24 (72.7)	
Rarely/never	24 (36.4)	6 (18.2)	
Condom use consistency with sex partner M ± SD	8.9 (3.2)	8.9 (5.3)	1.0
Condom use consistency with friends M ± SD	9.2 (3.4)	9.5 (5.2)	0.76
Condom use consistency with hook-up sex M ± SD	9.6 (3.4)	10.6 (5.3)	0.22
Power over condom use			0.36
Self	7 (10.8)	6 (18.2)	
Partner	18 (27.7)	11 (33.3)	
Both	23 (35.4)	12 (36.4)	
Neither	17 (26.2)	4 (12.1)	
Difficulty discussing condom use			0.55
Yes (difficult and very difficulty)	13 (20.0)	5 (15.2)	
No (Easy/very easy)	52 (80.0)	28 (84.8)	
Attitudes toward condom use^a^	28.1 (3.5)	29.5 (7.8)	0.20

Bold values indicate statistically significant.

^a^ Scale variable, 13 variables, range 1–52. Statistical test value *p* values: *t*-test was conducted for continuous variables and chi-square for categorical variables.

Participants with more than one sexual partner in the past year were more likely to use dating apps than those with only one partner (80.3% vs. 19.7%, *p* < 0.001). Similarly, participants who reported having more than three lifetime sexual partners were more likely to use dating apps than those with 1-2 lifetime sexual partners (72.7% vs. 27.3%, *p* < 0.001). There were no significant differences in condom use frequency during oral and vaginal sex between those who used dating apps and those who reported not using dating apps, except for anal sex condom use frequency. There was a significant difference in condom use among participants during anal sex when using dating apps (36.4% (never) vs. 28.8% (sometimes) vs. 34.8% (always), *p* < 0.001). Additionally, those who reported ever testing for HIV, frequent STI testing, and having sex while drinking alcohol were more likely to use dating apps than those who had never tested for HIV, infrequently tested for STI, or did not report sex while drinking alcohol.

Table 3 shows the results of multivariable Poisson regression modeling. Poisson regression analysis with robust variance showed that the probability of using dating apps was higher among those who resided in large towns [Prevalence Ratio (PR) = 1.42, 95% confidence interval (CI) = 1.05–1.91, *p* = 0.02], used HIV/STI screening services at the student clinic [PR = 1.58, 95% CI = 1.05–2.40, *p* = 0.03], ever tested for HIV and STI [PR = 1.54, 95% CI = 1.13–2.09, *p* = 0.01], and having more than one sexual partner in the past one year [PR = 1.54, 95%CI = 1.02–2.34, *p* = 0.04].). We checked for multicollinearity and based on the Variance Inflation Factor (VIF) results; multicollinearity was not a concern in the model. All VIF values were below 2, with a mean VIF of 1.38. Thus, the regression results did not have inflated variance due to correlated predictors.

**Table 3:** Multivariate poisson regression analysis with robust variance of dating app use and sexual behaviors among participants (*N* = 122).

Variables	Prevalence ratio (95% CI)	*P* value
Age		
18–24 (ref)	1.00	
25–35	0.94 (0.73, 1.22)	0.66
Gender identity		
Male (ref)	1.00	
Female	0.88 (0.64, 1.22)	0.46
Sexual orientation		
Heterosexual (ref)	1.00	
Gay/Lesbian/other identity	0.73 (0.45, 1.20)	0.21
Area of residence		
Small town (ref)	1.00	
Large urban	1.42 (1.05, 1.91)	**0**.**02**
Ever used HIV/STI screening at student clinic		
No (ref)	1.00	
Yes	1.59 (1.05, 2.40)	**0**.**03**
Sexual partners in the past 1 year		
0–1 (ref)	1.00	
>1	1.54 (1.02, 2.34)	**0**.**04**
Ever tested for HIV and STI		
No (ref)	1.00	
Yes	1.54 (1.13, 2.09)	**0**.**01**
STI testing frequency		
<6 months (ref)	1.00	
>=6months	1.30 (0.87, 1.94)	0.20
Sex while using alcohol		
No (ref)	1.00	0.25
Yes	1.15 (0.91, 1.45)	
Condom use attitude	1.00 (0.95, 1.03)	0.71

Bold values indicate statistically significant.

## Discussion

We examined the use of dating apps and their effects on condomless sexual behaviors among college students. Our study revealed that among college-aged young adults, the inclination to participate in sexual activities without using condoms and the convenience of finding sexual partners through dating apps may have detrimental effects on sexual behaviors, leading to a significant increase in the number of sexual partners. We found a relationship between the use of dating apps and an increased number of sexual partners among the participants. One notable observation was that the majority of participants who reported having two or more sexual partners demonstrated a higher likelihood of utilizing the campus health clinic for STI/HIV testing than those who reported having only one sexual partner. Some participants had undergone testing for STIs and HIV, while others disclosed having tested positive for a previous STI; however, most demonstrated inconsistent condom use. The university campus health clinic offers medical services to students and processes their insurance claims in a manner that is comparable to that of other healthcare facilities. Although STI and HIV testing is not a free service, the health clinic provides free STI and HIV testing services for all students periodically throughout the academic year. Nevertheless, the subsequent treatment and follow-up care are not provided at no cost to a student who tests positive for an STI.

### Use of dating apps

To date, the existing body of research has shown varying results regarding the potential adverse effects of dating apps on individuals’ sexual behavior. For example, one study found that dating app use was not directly related to STI diagnoses if users did not engage in condomless sexual practices (41). Additional research studies have discovered a correlation between active dating app usage, unprotected sexual behaviors, and STI acquisition (28, 29). There is a need for additional research to determine the complete scope of potential negative effects associated with dating app use. It is imperative to foster heightened awareness regarding the potential ramifications of these platforms on individuals’ engagement in condomless sexual behaviors as well as the transmission of STIs, including HIV infection. The results of our study align with previous scholarly works, suggesting that young adult populations require targeted STI/HIV prevention programs that are culturally sensitive and adapted to their specific needs.

### Implications

University campus health clinics play a vital role in providing STI/HIV testing services to students. Enhancing these services could involve expanding access to free testing or self-testing kits, increasing contraceptive availability, extending clinic hours, and offering “sex-positive” seminars and workshops (35, 37). Such efforts create inclusive, supportive learning environments and comprehensive health services, including free testing and treatment (35). Faculty support and administrative policy adjustments are crucial for implementing these programs successfully. Campus health clinics offer a unique opportunity to deliver accessible, confidential sexual health services that enhance student well-being (35–37). To optimize these efforts, universities should conduct environmental scans of existing services, collaborate with campus partners, implement evidence-based health campaigns, and explore alternative methods for distributing safer sex products, such as vending machines. These strategies could guide future steps in promoting sexual health on campuses.

We also discovered a positive element that could be connected to dating app use, such as using STI/HIV screening services at student health clinics and testing for STIs and HIV. Due to the possibility of engaging with students who utilize dating apps, all campus healthcare providers should adopt best practices to gather sexual health history from patients while avoiding stigmatizing behavior. Examples of this include using the 5 P's (38). Now may be an opportune time to explore systems-level change within these dating apps, such as incorporating STI risk and prevention information. An example is Grindr, an app that allows optional HIV disclosure (negative, negative, and PrEP, positive, positive with undetectable viral load). However, there have been concerns about the privacy and sharing of this data in the past (42). Additionally, there are numerous opportunities for misinformation and rapidly outdated data. Alternative approaches to disseminating STI/HIV status information through apps include implementing reminders for testing and screening through the application itself, collaborating with direct-to-consumer or self-sampling testing initiatives to improve screening rates, and assessing the feasibility of anonymous exposure notifications. Although there are currently several barriers, additional research should continue to investigate the effectiveness of app-based approaches in enhancing sexual health in this population. This is particularly important, considering that past studies have found that providing sexual health information on dating apps is widely accepted (30).

### Limitations and future directions

Several limitations should be considered when interpreting our findings. First, the use of self-reported data introduces the potential for recall bias, a common issue in cross-sectional studies. In addition, owing to the sensitivity of the topic, participants might have underreported condomless sexual behaviors (43), leading to an underestimation of the extent of actual unprotected sexual behaviors in this population. Third, the sample size was small compared with the total number of enrolled college students, and this study was conducted on one campus. Therefore, the findings of this study may not be generalizable to the broader college population. In addition, a small proportion of Black participants were included in this sample; thus, the findings may not be generalizable to Black students. Also, HIV and STI testing was measured as one variable, which limited our ability to disaggregate the results. We also did not collect information on participants’ fields of study, which limited our ability to include this variable in the regression model. Understanding participants’ fields of study could provide information on exposure and attitudes toward health education and sexual behaviors, particularly in relation to dating app usage. The field of study might influence knowledge about sexual health and practices, potentially affecting the way individuals engage with dating apps. Also including this data could enable more tailored interventions that address specific sexual behaviors associated with different academic fields. Future students should consider different strategies to increase Black student participation in research to assess condomless sexual behaviors and dating apps. Moving forward, future studies would benefit from implementing longitudinal designs. Future research should investigate the correlation between specific dating apps and unprotected sexual activities and explore potential techniques to effectively promote safe sex practices through customized messaging. Despite these limitations, the results of this study may inform the care provided on college campuses and guide sex-positive healthcare provider discussions regarding sexual behaviors associated with dating app use.

## Conclusions

This study highlights the significant association between dating app use and engagement in unprotected sexual behaviors among college students, including an increased number of sexual partners, inconsistent condom use, and the potential for STI and HIV transmission. While dating apps provide opportunities for social interaction and sexual exploration, they also pose risks, particularly for young adults navigating their sexual health. Our findings align with existing research suggesting the need for targeted STI/HIV prevention programs that address the specific needs and contexts of college students. University health clinics play a critical role in providing accessible and confidential sexual health services, including STI/HIV testing, education, and prevention initiatives. Enhancing these services through expanded access, supportive environments, and strategic partnerships can help mitigate the risks associated with dating app use. Additionally, integrating sexual health information and reminders within dating apps could offer innovative ways to promote safer sexual practices.

Future research should continue to explore the impact of dating app use on sexual health, particularly through longitudinal studies that examine specific app usage patterns and their effects over time. Further investigation into app-based interventions and their effectiveness in promoting sexual health will also be crucial. Understanding these dynamics will help develop comprehensive strategies to support the well-being of college students in the evolving digital landscape.

## Data Availability

The original contributions presented in the study are included in the article/Supplementary Material, further inquiries can be directed to the corresponding author.
